# Comparing the power of family-based association tests for sequence data with applications in the GAW18 simulated data

**DOI:** 10.1186/1753-6561-8-S1-S27

**Published:** 2014-06-17

**Authors:** Jing Huang, Yong Chen, Michael D Swartz, Iuliana Ionita-Laza

**Affiliations:** 1 Division of Biostatistics, University of Texas School of Public Health, Houston, TX 77030, USA; 2 Department of Biostatistics, Columbia University, Mailman School of Public Health, New York City, NY 10032, USA

## Abstract

We apply a family-based extension of the sequence kernel association test (SKAT) to 93 trios extracted from the 20 pedigrees in the Genetic Analysis Workshop 18 simulated data. Each extracted trio includes a unique set of parents to ensure conditionally independent trios are sampled. We compare the empirical type I error and power between the family-based SKAT and the burden test under varying percentages of causal single-nucleotide polymorphisms included in the analysis. Our investigation using simulated data suggests that, under the setting used for Genetic Analysis Workshop 18 data, both the family-based SKAT and the burden test have limited power, and that there is no substantial impact of percentage of signal on the power of either test. The low power is partially a result of the small sample size. However, we find that both the family-based SKAT and the burden test are more powerful when we use only rare variants, rather than common variants, to test the association.

## Background

Genome-wide association studies (GWAS) have proven to be a powerful approach to identify novel common single-nucleotide polymorphisms (SNPs) contributing to the etiology of complex traits [1]. However, identifying rare genetic variants with minor allele frequency (MAF) <5% that are associated with complex diseases remains challenging. Standard statistical tests for common variants (MAF >5%) are underpowered for rare variants because of their low frequencies and moderate effect sizes. Even with appropriate methods, larger sample sizes are required to have variation in the rare variants [2,3].

A major limitation of population-based association analyses is the potential for unrecognized population heterogeneity as a result of population stratification. This problem, however, can be well addressed through the use of family-based studies, which use related individuals in association studies. Family-based controls eliminate the need to adjust for population structure [4,5]. Another advantage of using family-based controls is the ability to identify and correct technological artifacts in the data, investigations of questions such as parent-of-origin effects and other applications that are imperfectly or not readily addressed in case-control association studies [4,5].

The data set for the Genetic Analysis Workshop 18 (GAW18) consists of whole genome sequence data from a pedigree-based sample. These pedigrees are drawn from the Type 2 Diabetes Genetic Exploration by Next-generation sequencing in Ethnic Sample Project 2 (T2D-GENES Project 2). The T2D-GENES Project 2 is designed to identify low-frequency or rare variants influencing susceptibility to type 2 diabetes using information from whole genome sequencing of 1043 individuals from 20 Mexican American pedigrees enriched for type 2 diabetes from San Antonio, Texas. The pedigree data are drawn from 2 San Antonio-based family studies: the San Antonio Family Heart Study (SAFHS) and the San Antonio Family Diabetes/Gallbladder study (SAFDGS).

A variance component test, known as a sequence kernel association test (SKAT), is proposed for testing associations of rare variants in population-based designs [2,6]. SKAT is shown to be powerful when rare variants have effects in different directions, and it is computationally efficient because of the simple limiting distribution of the test statistic. However, SKAT is designed for testing associations in unrelated subjects and cannot be directly applied to family-based designs. Some investigators have proposed an extension of SKAT to family-based designs [7], hereafter referred to as family-based SKAT. In this report, we apply it to the GAW18 simulated data and explore more features of the test statistics.

## Methods

The data set for GAW18 includes 959 individuals out of 1043 individuals from 20 Mexican American pedigrees of the T2D-GENES Project 2. We conducted our analysis using the simulated phenotypes, baseline systolic blood pressure (SBP) and diastolic blood pressure (DBP), and the whole genome sequenced and imputed genotypes of the 959 correlated individuals. To keep the notation simple and make our discussion transparent, we considered trio designs, acknowledging the fact that the method is applicable to more general family structures.

### Trio selection

Conditionally independent trios were extracted from the 20 extended pedigrees. Each extracted trio included a unique set of parents. For nuclear families with more than 1 offspring, we randomly selected 1 offspring and formed a trio with the parents. Specifically, the individuals were grouped into families by the parents' identifications, and 1 offspring was selected with equal probability to form a trio. We only selected trios that had complete genotype data for all 3 family members. Finally, 93 conditionally independent trios were extracted from the GAW18 data.

### SKAT and burden test for family-based design

The family-based SKAT proposed recently [7] can be described as follows. For the *i*th trio, denote the specific region of the genome by G, the offspring trait by $Y_{i}$ and the offspring genotype at the *j*th variant in G by X*_ij _*(1 ≤ j ≤m), where *m *is the number of variants in the region G. We assume a generalized linear mixed effects model (GLMM) as follows: $h\left[\mu_{i}\right]=C_{i}\alpha +X_{i}\beta$, where $\mu_{i}=E\left(Y_{i}\right)$, h(.) is a known link function, *α *is the regression coefficients for the potential confounders $C_{i}$, and *β *is the vectors of regression coefficients for the m variants $X_{i}$ , respectively. It is further assumed that the coefficients, $\beta_{j}$s, are independent random variables and follow an unspecified distribution with mean 0 and variance $w_{j}^{2}\tau$. Here $w_{j}$ can be considered as a weight that can be a function of the data (such as genotype frequencies estimated from the parents) or externally defined (such as a functional prediction score). Under the GLMM assumption, testing the null hypothesis of no genetic effect, that is, all *β*s equal to 0, is equivalent to testing $H_{0}:\tau =0$, that is, nonexistence of the variance component in the GLMM. Similar to SKAT, the score test for a family-based design is $Q_{S}={\left(Y-{\hat{\mu }}_{0}\right)}^{T}\tilde{K}\left(Y-{\hat{\mu }}_{0}\right)$, where ${\hat{\mu }}_{0}=C\hat{\alpha }$ for continuous traits, ${\hat{\mu }}_{0}=logit^{-1}\left(C\hat{\alpha }\right)$ for dichotomous traits, and $\tilde{K}=\left[X-E\left(X|X_{p}\right)\right]WW{\left[X-E\left(X\text{|}X_{p}\right)\right]}^{T}$ is a weighted linear kernel. For the kernel, *X *represents the offspring genotype matrix, $X_{p}$ represents the parental genotype matrix, and $W=\text{diag}\left(w_{1},\ldots ,w_{m}\right)$ represents variant weights based on parental genotypes. In this study, we define $w_{j}=Beta\left({\hat{f}}_{j};a,b\right)$, where ${\hat{f}}_{j}$ is is the estimated variant frequency based on parental genotypes. Under the null hypothesis, $E\left(X|X_{p}\right)$ can be calculated using the laws of mendelian transmission. For the linear kernel, $Q_{S}$ has a simple expression: $Q_{S}=\overset{m}{\underset{j=1}{\sum }}w_{j}^{2}{\left[\overset{N}{\underset{i=1}{\sum }}\left(Y_{i}-{\hat{\mu }}_{i,0}\right)\left(X_{ij}-E\left(X_{ij}\text{|}X_{ij}^{P}\right)\right)\right]}^{2}$, where $X_{ij}^{P}$ is the parental genotype data for family *i *at variant *j*. It can be shown that the test statistic $Q_{S}$ has a limiting distribution of a mixture of chi-square distributions. Specifically, $Q_{S}$ converges weakly to $\overset{m}{\underset{j=1}{\sum }}\lambda_{j}\chi_{1,j}^{2}$, where ($\lambda_{1},\ldots ,\lambda_{m}$) are the eigenvalues of matrix $A^{1/2}L^{T}WWLA^{1/2}$, with $LAL^{T}=Cov\left({\left(X-E\left(X\text{|}X_{p}\right)\right)}^{T}\left(Y-X\hat{\alpha }\right)|X_{p},Y\right)$. Originally, the family-based SKAT assumes that all *β *coefficients are independently distributed. To allow for possible correlation of effects among different variants, a family kernel was proposed [2]: $\tilde{K}=\left[X-E\left(X|X_{p}\right)\right]WR_{\rho }W{\left[X-E\left(X\text{|}X_{p}\right)\right]}^{T}$, where $R_{\rho }=\left(1-\rho \right)I+\rho 11^{T}$ specifies an exchangeable correlation matrix. The test statistic is $Q_{\rho }={\left(Y-{\hat{\mu }}_{0}\right)}^{T}{\tilde{K}}_{\rho }\left(Y-{\hat{\mu }}_{0}\right)$. When ρ = 0, $Q_{\rho }$ equals $Q_{s}$, where all *β *coefficients are assumed independent. When ρ = 1, the test statistic becomes $Q_{\rho }={\left[\overset{m}{\underset{j=1}{\sum }}w_{j}^{2}\overset{N}{\underset{i=1}{\sum }}\left(Y_{i}-{\hat{\mu }}_{i,0}\right)\left(X_{ij}-E\left(X_{ij}\text{|}X_{ij}^{P}\right)\right)\right]}^{2}$, which is equivalent to the test statistics in the family-based association test (FBAT) [8]. The *p *value was calculated using the moment matching approach [9] or inverting the characteristic function [10], as considered by Lee et al [11].

### Analysis strategy

The goal of our analysis was to assess the power of detecting association between the simulated quantitative phenotypes (baseline SBP and DBP) and the causal genes (from the simulation answer sheet) on chromosome 3 by the family-based SKAT and the burden test, whether or not adjusting for different proportions of causal variants. To ensure a fair comparison of power, the empirical type I error rates of all tests were evaluated by using the variable Q1 (a quantitative trait in the simulation data set, simulated to be not associated with any of the SNPs). To evaluate the power of tests, we conducted the family-based SKAT and the burden test for each causal gene using, respectively, baseline SBP and DBP as the response trait. Each of the 2 tests was conducted separately by including rare variants only, common variants only, and all variants of each gene. Therefore, 12 tests were conducted at each gene. Table 1 describes the scenarios of the tests. The proportion of causal variants among all variants in each gene (referred to as strength of signal) may have impact on the power of tests. To adjust for that proportion, we conducted an analysis similar to the analysis in the unadjusted model under varying proportions of the causal SNPs (10%, 25%, and 50%). Then we performed another 36 analyses to compare the power of tests. Considering that the effect size of causal SNPs differs across SNPs, we fixed the causal SNPs included in all the scenarios, but diluted the strength of signal by including differing numbers of noncausal SNPs that are randomly chosen from each gene to construct the proportion. For consistency and to prevent a proportion of 0 signal, we included only causal genes with at least 1 causal rare variant and at least 1 causal common variant. Each analysis was conducted using all 200 simulated data sets.

**Table 1:** Scenarios of the 12 tests performed in comparing family-based SKAT and burden test using different types of variants (i.e., common vs. rare) and different types of outcome (i.e., DBP vs. SBP)

	Outcome		Approach		Variants included	
			
	DBP	SBP	Family SKAT	Family burden	Rare variants	Common variants
Test 1	√		√		√	
Test 2	√		√			√
Test 3	√		√		√	√
Test 4	√			√	√	
Test 5	√			√		√
Test 6	√			√	√	√
Test 7		√	√		√	
Test 8		√	√			√
Test 9		√	√		√	√
Test 10		√		√	√	
Test 11		√		√		√
Test 12		√		√	√	√

### Power comparisons

For the analysis without adjusting for proportion of causal variants in the gene, we used the generalized estimating equation (GEE) [12] method to test for the differences in power between scenarios, accounting for the correlations induced by analyzing the same gene 12 times. Specifically, of the 200 simulations, let Y*_ij _*denote the number of successful rejection of the null hypothesis for the *j*th test of the *i*th gene, and $p_{ij}$ the estimated power for each test, *i *= 1,2,...31, *j *= 1,2,...12. We treated the Y*_ij_*s as correlated measures for the *i*th *gene*, and then we constructed a binomial regression model using GEE method to compare the power for each test:

$$y_{ij}\sim Binomial\left(200,p_{ij}\right)$$

$$\begin{array}{c} logit\left(p_{ij}\right)=\beta_{0}+\beta_{1}I\left(SBP_{ij}\right)+\beta_{2}I\left(common_{ij}\right)+\beta_{3}I\left(common\;and\;rare_{ij}\right)\\ \;+\beta_{4}I\left(SKAT_{ij}\right) \end{array}$$

where $\beta_{1}$ represents the difference in power for using SBP rather than DBP as the outcome, $\beta_{2}$ represents the difference in power for using common variants instead of rare variants, $\beta_{3}$ represents the difference in power for jointly using common and rare variants compared with using rare variants only, and $\beta_{4}$ represents the difference in power for using the family-based SKAT rather than the family-based burden test. Here I(A) denotes the indicator function, which equals 1 when A is true and 0 otherwise. Additionally, these effects are evaluated in similar model adjusting for the proportion of causal variants in the gene where *j *= 1,2,...,36.

## Results

### Trio and causal SNPs

Using the approach stated in the methods section, we extracted a total of 93 trios from the GAW18 data. Our analysis focuses on chromosome 3 only. With knowledge of the simulating model, the 31 causal genes were available for the family-based SKAT and the burden test of all SNPs. When examining different power to detect the association under different proportions of causal SNPs, only the 16 causal genes that contain at least 1 causal rare variant and at least 1 causal common variant were included.

### Gene-based test of all SNPs

We applied the family-based versions of the burden and SKAT tests on the 93 trios for the gene-based association test of the 31 causal genes, using the 200 simulated data sets. Variable Q1 (a quantitative trait in the simulation data set that is not associated with any of the SNPs) was used to test for type I error and the empirical type I error rates are close to the nominal level of 0.05 with a range of (0.043 to 0.059), which is within the 95% confidence interval of the nominal level, that is, (0.035, 0.065). An earlier study [7] also reported that false-positive rates of the methods we applied were well controlled using large simulations with considerable sample size. Consequently, we used 0.05 as the critical value when calculating power.

**Figure 1:**
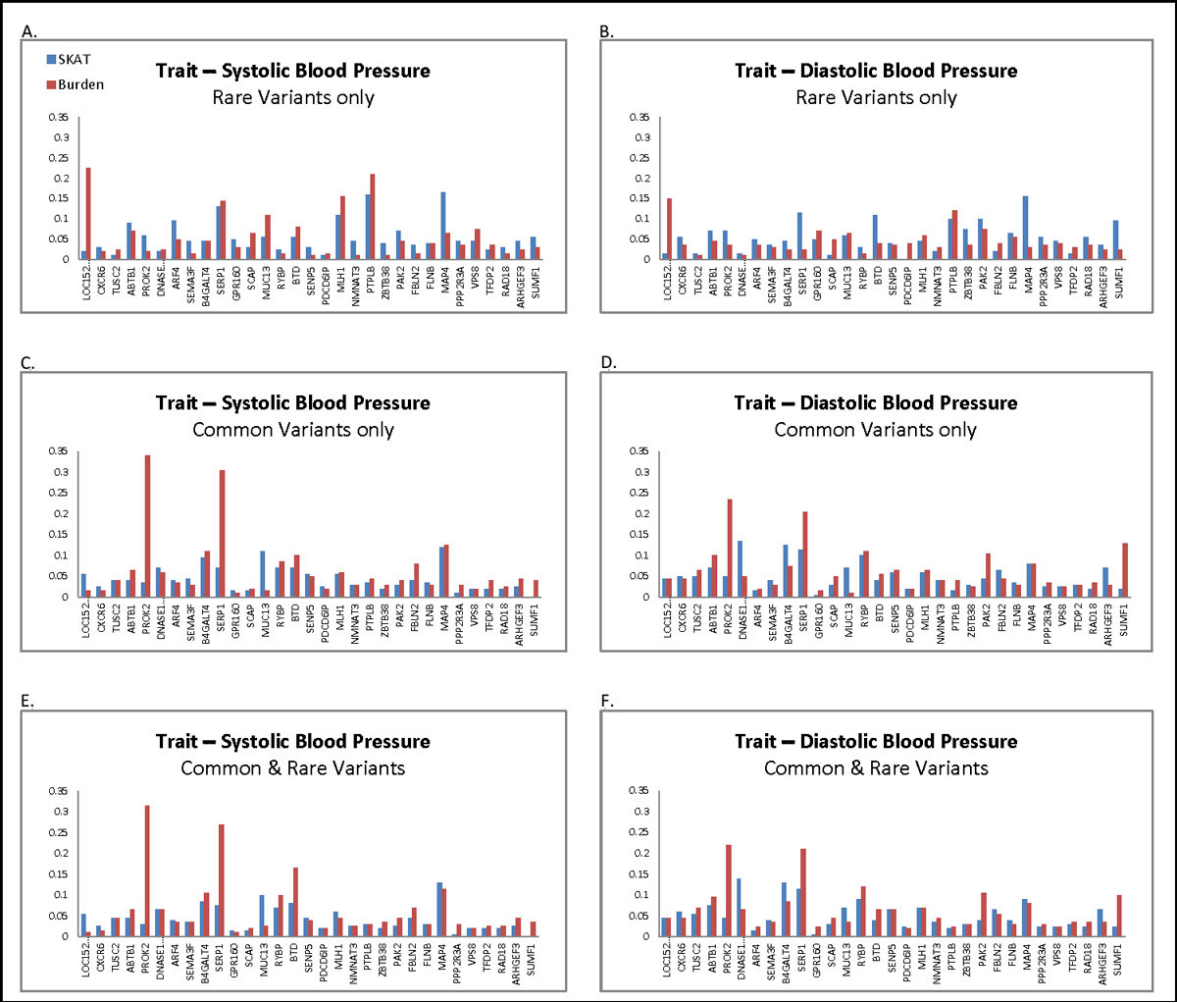
**Power of tests using all available SNPs in the data (α = 0.05)**. Plots in the left panels use SBP as a continuous outcome. Plots in the right panels use DBP as a continuous outcome. All 6 plots use the same legend. Plots in the first row use SNPs with MAF *≤*0.05. Plots in the second row use SNPs with MAF >0.05. Plots in the third row use both.

Figure 1 shows the power of correctly identifying causal genes at the α = 0.05 level. Plots in the left-side panels show similar patterns to those in the right-side panels, which is not surprising considering that SBP and DBP are highly correlated phenotypes. According to the simulating model, gene *MAP4 *has a strong signal. Our results show both the family-based SKAT and the burden test are able to detect *MAP4*.

In Figure 1C to F, we observed 2 peaks in the plots, which are the results of genes *PROK2 *and *SERP1*. For both genes, the peaks were observed only when common variants were included in the test. This finding is consistent with the underlying simulating model, in which almost all the causal SNPs in these 2 genes are common variants. However, the family-based SKAT did not show any power beyond type I error to identify these two genes.

### Testing under different proportions of causal SNPs

**Figure 2:**
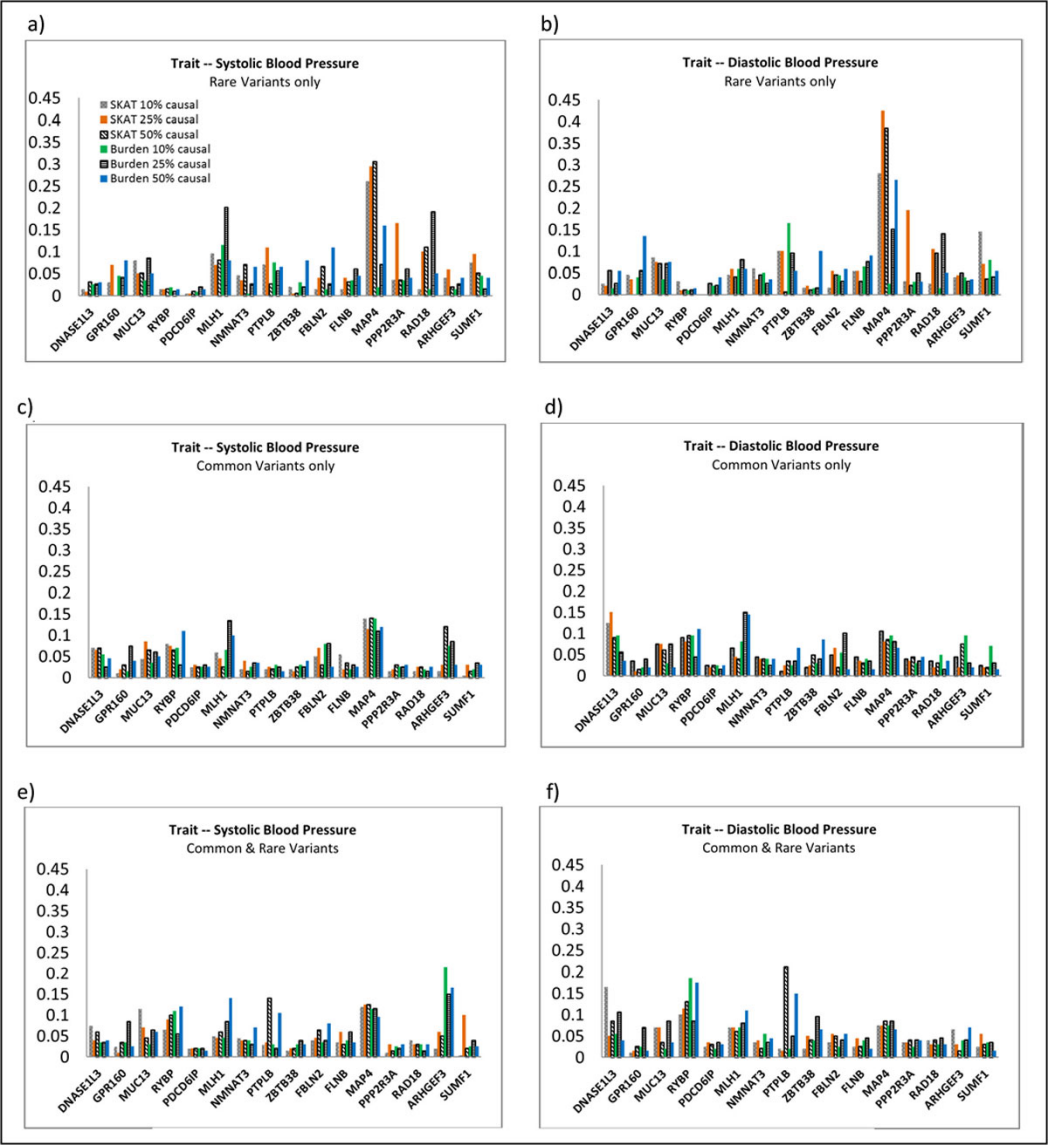
**Power of tests under different proportions of causal SNPs (α = 0.05)**. Plots in the left panels use SBP as a continuous outcome. Plots in the right panels use DBP as a continuous outcome. All 6 plots use the same legend. Plots in the first row use SNPs with MAF *≤*0.05. Plots in the second row use SNPs with MAF >0.05. Plots in the third row use both.

We conducted both the family-based SKAT and the burden test in scenarios containing different proportions (ie, 10%, 25%, 50%) of causal SNPs in the analysis. Figure 2 shows the results.

We did not observe a substantial impact of percentage of causal SNPs on the power of both tests from the plot, whether or not the analysis included rare variants. The power of both the family-based SKAT and the burden test is comparable across most genes. Two prominent exceptions are the genes *MAP4 *and *MLH1*. In Figure 2A, the family-based SKAT has much higher power than the burden test when using rare variants only in gene *MAP4*. The possible explanation is that the causal rare variants in *MAP4 *affect SBP in different directions (also confirmed by the simulating model), thus the burden tests lose considerable power because causal variants β coefficients are in mixed directions. When we included common variants in the analysis, the power of the family-based SKAT decreased. This is a result of very few common SNPs being causal in *MAP4*; therefore, adding common variants increases the number of noncausal SNPs and dilutes the causal signals. The burden test, however, had a slightly higher power than the family-based SKAT in gene *MLH1*. Examination of the simulating model suggests that almost all variants in *MLH1 *affect DBP and SBP in the same direction, so it is not surprising that the burden tests had comparable or better performance than the family-based SKAT for testing the variants in *MLH1*.

Another interesting peak was observed at gene *PTPLB1 *(Figure 2E and 2F) when using 50% causal SNPs and both common and rare variants. Both the family-based SKAT and the burden test showed higher power than other scenarios. The power is lower for testing either rare variants only or common variants only, which suggests that combining rare variants and common variants together may increase the power of both tests.

### Power comparisons

In addition to visually comparing power as presented in Figures 1 and 2, we used GEE methods to more rigorously compare the power under different scenarios. Specifically, we did not detect significant differences (*p *value >0.3) in power between the family-based SKAT and the burden test across all scenarios, whether we adjusted for the proportion of causal variants or not. However, after adjusting for proportions of causal variants, we found that on average, the tests using common variants only had less power compared to those using rare variants only, followed by the tests using both common and rare variants. The test for overall difference in power yields a *p *value of 0.04.

## Discussion

Our analysis using the GAW18 simulated baseline phenotypes and sequence genotypes with sample size of 93 conditionally independent trios shows limited power of both the family-based SKAT and the burden test. The low power is most likely the result of using a small number of trios and the weak signals in the simulating model. However, we found that both models adequately controlled the type I error rates with only 93 trios. This agrees with the results of simulation studies in [7], where a large number of trios are considered (n = 500). Furthermore, after adjusting for proportion of causal variants, we found significant differences in power between tests using common variants only versus tests using rare variants only or both common and rare variants. Larger number of trios are needed to confirm this finding as suggested by [13,14].

Recently, 2 methods using SKAT for family data have been proposed [15,16]. Both of these methods take into account the whole family structure by using a marginal model with correlation structure specified by kinship matrix. However, there is a subtle difference between these 2 methods and our method. These 2 methods are comparing allele frequencies as a population-based test using the mixed-modeling framework to take into account the correlation among the individuals within a family, whereas our method is a transmission disequilibrium type (TDT) test, which is conditioned on parental genotypes and compares allelic transmissions. In the absence of population structure, the population-based association tests using the whole family are expected to be more powerful than our method. However, in the presence of population structure, the former tests may lead to inflated type I errors whereas our method is robust to population structure. Hispanic populations, such as the one used in this study, are likely to be admixed [17] and, therefore, the TDT-based method remains robust to potential population structure.

## Competing interests

The authors declare that they have no competing interests.

## Authors' contributions

Jing Huang and Yong Chen drafted the manuscript. Jing Huang conducted statistical analyses. All authors revised, read, and approved the final manuscript.
